# Current and future therapeutic options for chronic hepatitis D virus infection

**DOI:** 10.3389/fcimb.2024.1382017

**Published:** 2025-02-11

**Authors:** Mariantonietta Pisaturo, Antonio Russo, Pierantonio Grimaldi, Salvatore Martini, Nicola Coppola

**Affiliations:** Department of Mental Health and Public Medicine, Section of Infectious Diseases, University of Campania Luigi Vanvitelli, Naples, Italy

**Keywords:** HDV, HDV management, HDV treatment, bulevirtide, lonafarnib

## Abstract

In the last few years there have been innovations in HDV therapy which have brought new excitement in the scientific community also considering the few therapeutic opportunities. Recently, new molecular targets have been identified, both in monotherapy and in combination with peginterferon alpha (PegIFNα). Evaluating this review of the literature of the last ten years, HDV-related chronic hepatitis seems to have become a potentially curable disease, a statement that was unthinkable a few years ago. There are old and new weapons at our disposal. The old weapons are PegIFNα and recently PegIFN-lambda (PegIFNλ). PegIFNα, for which there are more data, appears to be an excellent combination regimen, if not contraindicated, both for Bulevirtide (BLV), data supported by important clinical trials and real-world studies, and probably for lonarfanib, although in the latter case the results are not yet definitive as the studies are fewer. However, data on long-term follow-up are needed.

## Introduction

Hepatitis delta virus (HDV) is the smallest known human virus with a ~1.7 kb RNA genome; it is a defective RNA virus which depends on hepatitis B virus surface antigen (HBsAg) for the assembly of new infectious virions.

HDV has a simple structure, encodes for the only one known viral protein, the hepatitis delta antigen (HDAg), which occurs in two different forms: the small HDAg (24kDa) which is important for virus replication, and the large variant (27kDa) generated by a post-transcriptional RNA-specific adenosine deaminase-mediated RNA-editing event, which is able to inhibit replication and promote virion assembly (Wang et al., 1986; Chang et al., 1991). The two HDAg proteins bind to the HDV RNA genome to form a ribonucleoprotein which is then surrounded by an envelope containing the three isoforms of HBsAg (Large-HBsAg, Medium-HBsAg and Small-HBsAg). HDV binds to the same cellular receptor as hepatitis B virus (HBV), the sodium taurocholate cotransporter polypeptide (NTCP), by means the interaction with the pre-S1 domain of the Large-HBsAg isoform, thereby mediating HDV entry into hepatocytes (Hourioux et al., 1998; Yan et al., 2012).

HDV RNA acts as a ribozyme and cleaves to replicate; it does not encode proteins with enzymatic activity and so HDV borrows the enzymes necessary for replication from the infected cell. The balance between viral replication and assembly is conducted by the ratio of small and large HDAg and by different post-translational modifications such as prenylation, phosphorylation, methylation, acetylation and sumoylation (Hourioux et al., 1998; Huang et al., 2006).

Acute HDV infection occurs either from simultaneous coinfection with HBV, which has a chronicity rate less than 17% or from superinfection of patients with chronic HBV infection, which has a higher chronicity rate, approximately 80% (Yurdaydın et al., 2010; Farci and Niro, 2012).

Chronic hepatitis D (CHD) is associated with a worse clinical outcome than HBV mono-infection resulting in cirrhosis in 15% of cases in 1-2 years and in 70-80% of cases in 5-10 years of follow-up; furthermore, rates of hepatocellular carcinoma and of hepatic decompensation are 2-3 times higher than in HBV monoinfection (Fattovich et al., 1987; Romeo et al., 2009; Niro et al., 2010; Yurdaydın et al., 2010). However, the natural history of CHD today appears more benign than previously thought (Kamal et al., 2023). This consideration, however, is i) true in Western countries; ii) derives from heterogeneous studies with limited numbers of patients and iii) it is the result of the improvement in therapeutic management and supportive care of CHD and the improvement in the management of viral co-infections and comorbidities (Kamal et al., 2023). Although the natural history seems to be more benign than expected, patients with CHD have a worse prognosis than patients with chronic hepatitis B (CHB) alone, given the high rate of liver complications (cirrhosis, cirrhotic decompensation, hepatocellular carcinoma) (Kamal et al., 2020; Fattovich et al., 2000; Miao et al., 2020). Moreover, today the most important negative prognostic factor, HDV RNA positivity, has been identified. Patients with detectable viremia have a 3.8-fold increase in presenting hepatic complications compared to anti-HDV-positive but non-viremic patients (Kamal et al., 2020; Da et al., 2019; Miao et al., 2020).

The international epidemiology of HDV is challenging to accurately estimate due to limited active surveillance for this rare infectious disease (Sagnelli et al., 2021; Pisaturo et al., 2023; Razavi et al., 2023). Three recent meta-analyses show different estimates of HDV prevalence. Chen et al. estimated an HDV prevalence in the general population of 0.98% (95% confidence interval (CI) 0.61 to 1.42) and of 14.57% in the pooled HBsAg-positive population (95% CI 12.93 to 16.27) worldwide, with a high end estimation of 72,451,000 anti-HDV-positive subjects (Chen et al., 2019). Stockdale et al. calculated an anti-HDV prevalence of 0.16% (0.11-0.25) in the general population, of 4.5% (95% CI 3.6-5.7) among all HBsAg-positive subjects and of 16.4% (14.6-18.6) among those attending hepatology clinics, with an estimation of 12,000,000 anti-HDV-positive subjects (Stockdale et al., 2020). Miao et al. reported that the pooled prevalence of HDV was 0.80% (95% CI, 0.63-1.00) in the general population and 13.02% (95% CI, 11.96-14.11) in HBV carriers, with an estimation of 48,000,000 to 60,000,000 anti-HDV-positive subjects (Miao et al., 2020). Results from collaborators at the Polaris Observatory have re-evaluated the data using a different approach and in most of the 25 countries evaluated (18/25) have identified a lower HDV prevalence compared to previous data (Polaris Observatory Collaborators, 2023). More recent epidemiologic studies suggest a prevalence in the US hepatitis B population closer to 3-5% (https://www.who.int/news-room/fact-sheets/detail/hepatitis-d; https://www.cdc.gov/hepatitis/hdv/hdvfaq.htmsection1; https://www.cdc.gov/hepatitis/global/index.htm; https://www.aasld.org/liver-fellow-network/core-series/clinical-pearls/hepatitis-d-mystified; Asselah and Rizzetto, 2023; Pearlman, 2023).

Due to HDV simplicity in structure and the lack of its own viral polymerase, it is a challenge to identify HDV specific targets for antivirals. Standard HBV DNA polymerase nucleos(t)ide analogue-based (NUC) therapeutics are mostly ineffective for HDV treatment. Thus, therapeutic options for HDV were very limited up to a few years ago. In fact, until recently, standard interferon (IFN) alpha and its pegylated form (PegIFN) were the only treatment options for CHD.

The specific mechanism of action of IFNα on HDV is not clear. *In vitro* studies suggest that IFN marginally inhibits HDV replication in stably infected cells (Ilan et al., 1992; Zhang et al., 2018). Recent studies have also showed that both IFNα and IFN lambda (IFN λ) significantly reduced HDV infection when given at an early stage of the infection, suggesting an inhibitory effect on viral entry (Zhang et al., 2018). Furthermore, both IFNs suppress HDV spread that is mediated by cell division, possibly by increasing the elimination of HDV replicative intermediates during mitosis (Zhang et al., 2022).

The most important studies on IFN α for HDV treatment are two randomized controlled trials, Hep-Net–International Delta Hepatitis Intervention Trial (HIDIT I) HIDT I and Hep-Net–International Delta Hepatitis Intervention Trial II (HIDT II) (Wedemeyer et al., 2011; Wedemeyer et al., 2019b) and many uncontrolled trials with prospective and retrospective designs. Recently the results of these studies were summarized in a meta-analysis showing that, at the end of a 24-week post-treatment, the pooled virological response was achieved in 29% (95% CI: 24%-34%) (Abdrakhman et al., 2021). Moreover, the combination of PegIFNα-2a with adefovir for 48 weeks or with tenofovir disoproxil fumarate (TDF) for 96 weeks did not significantly improve the off-treatment virological responses (Wedemeyer et al., 2011; Wedemeyer et al., 2019b).

Furthermore, other studies suggested that more than 50% of patients with a virologic response at 24 weeks post-treatment developed virological relapse later, up to 10 years after the end of IFNα treatment (Heidrich et al., 2014; Wranke et al., 2020). According to these results the efficacy of IFN-based treatment for HDV is very poor.

Furthermore, IFNs have limited use in clinical practice given that this drug is contraindicated in elderly people or in those with autoimmune and/or psychiatric diseases or with advanced or decompensated liver disease (European Association for the Study of the Liver, 2017).

In the last few years there have been innovations in HDV therapy which have brought new excitement to the scientific community, also in consideration of the previous few therapeutic opportunities available. In fact, new molecular targets have recently been identified, prescribed as monotherapy or in combination with PegIFNα have been evaluated in clinical trials (Urban et al., 2021). Among them, the peptide entry-inhibitor bulevirtide (BLV), which blocks the binding of HBsAg-enveloped particles to the NTCP (sodium taurocholate co-transporting polypeptide), which is the cell entry receptor for both HBV and HDV, preventing the entry of HDV into hepatocytes and subsequent spreading of the virus (Tu and Urban, 2018). In July 2020, BLV 2 mg received conditional marketing authorization by the EMA (European Medicines Agency) for the treatment of CHD, with the recommendation to maintain the treatment until clinical benefit is observed (https://www.ema.europa.eu/en/medicines/human/EPAR/hepcludexauthorisation-details-section). Moreover, other molecules are currently under investigation to improve the therapeutic management of CHD.

The aim of the present narrative review was to show the recent data relating to HDV therapy, and to analyze the future therapeutic options for this severe chronic infection.

## Antiviral treatment monitoring and endpoints

Before analyzing the treatment options for CHD, it seems useful to evaluate the monitoring and determination of the end-points for the antiviral therapy. The ideal end-point for every antiviral regimen in CHD would be to obtain the HBsAg loss, which, however, occurs only in 2.5% of cases treated with Peg-IFN (Yurdaydin et al., 2018b; Abdrakhman et al., 2021). Thus, we have to settle for other realistic end-points.

In the case of therapy with PegIFNα, the end-point is negativity of HDV RNA 24 weeks after stopping treatment (Sustained virological response-SVR). This occurs in 29% of cases (Yurdaydin et al., 2018b; Abdrakhman et al., 2021); however, as reported above, the relapse of HDV infection is subsequently frequent, occurring in 50% (Wranke et al., 2020) of cases 5 years after the end of treatment and in 57% at 8.9 years (Wranke et al., 2020). In regards to monitoring, in Peg-IFN-based treatment, HDV RNA should be tested every three to six months during therapy, at the end of treatment and subsequently, in the case of a virological response, it should be repeated 6 and 12 months after the end of the treatment and then every year. In addition to HDV RNA monitoring, testing of HBV DNA and HBsAg should be done at the same time intervals. It is also necessary to continue with ultrasound surveillance to exclude the development of hepatocellular carcinoma (European Association for the Study of the Liver, 2023).

Establishing end-points during therapy with new drugs against HDV is even more complex. For the Food and Drug Administration (FDA), a reduction of ≥2 logs compared to baseline in the HDV load with a normalization in transaminases should be achieved (Food and Drug Administration, Center for drug evaluation and research (CDER), 2019); for the European Association for the Study of the Liver (EASL), American Association for the Study of Liver Diseases (AASLD), the end-point of treatment should be HDV RNA negativity at week 48 of treatment (Cornberg et al., 2019; European Association for the Study of the Liver, 2023). This is an important point, considering that the ideal endpoint should be considered the sustained HDV RNA negativity. As regards the monitoring, HDV-RNA should be monitored every 3-6 months during BLV treatment, and in the case of BLV discontinuation, it should be tested at the time of treatment discontinuation, after 1, 3, 6, 12 months and yearly thereafter to monitor for relapse of viral replication. HBsAg testing should be performed every year during and after therapy.

## Interferon-based therapies

In the last 30 years standard IFNα or its pegylated form were the only treatment option in patients with CHD. The latest European guidelines confirmed IFNα, especially the pegylated form, as one of the two drugs that can control CHD (European Association for the Study of the Liver, 2023). IFN was discovered in 1957 by Alick Isaac and Jean Lindenmann, who identified this substance as interfering with the activity of the influenza virus in agglutinating red blood cells (Sandmann and Wedemeyer, 2023). The antiviral activity of IFN has not yet been fully elucidated, but research has shown that IFN-stimulated genes (ISGs) can inhibit viral attachment, entry and trafficking as well as viral gene expression, viral protein translation, viral genome amplification, viral particle assembly and egress (Schoggins, 2019). The latest European guidelines consider the use of Peg-IFNα in all patients with CHD and compensated liver disease and prefer a 48 week regimen considering the patient’s clinical condition, treatment tolerance and virologic response through HDV RNA and HBsAg (European Association for the Study of the Liver, 2023).

In the last 10 years, from 2013 to 2023, fourteen original articles have investigated the efficacy of IFNα in patients with CHD (Anastasiou et al., 2024; Karaca et al., 2013; Abbas et al., 2014; Heller et al., 2014; Keshvari et al., 2014; Bahcecioglu et al., 2015; Abbas et al., 2016; Borzacov et al., 2016; Niro et al., 2016; Soyer et al., 2016; Wranke et al., 2017; Boglione et al., 2019; Wedemeyer et al., 2019b; Etzion et al., 2023), both clinical trials and real-life studies.

### Clinical trials on PegIFNalpha-based therapy

**Table 1** shows the data from the clinical trials. They suggested that the addition of a nucleos(t)ide analogue to PegIFN-α added no antiviral effect. In fact, HDV RNA undetectability at the end of follow up (EOFU), 24 weeks after stopping PegIFN-alpha of each study ranged from 16% to 52.6%, with ALT normalization ranging from 19% to 46% with PegIFNα monotherapy, and from 18% to 75% and 19%-36%, respectively, with combination therapy (**Table 1**). Only two studies evaluated HBsAg loss at EOFU, an event not frequent but possible (**Table 1**).

**Table 1:** Clinical studies investigating the impact of Peginterferon in HBV/HDV infection.

Authors,years	Type of study	Enrollment period	Sample size	Age at enrollment (mean and range)	Follow up	Interventional group treatment	Control group treatment	HBsAg loss at the EOFU*	AST/ALT Normalization at the EOFU*	HDV-RNA Undetectability at the EOFU*	Adverse drug reaction leading discontinuation of IFN** treatment
(**Heller et al., 2014**)	Clinical trial (Phase 2)	2002-2006	13	42 (18–58)	260 weeks	PEG-IFN^£^ alfa 180 mcg	–	3(25%)	3(25%)	3(25%)	3 patients required treatment discontinuation, two (15%) of whom were possibly related to peginterferon
(**Abbas et al., 2016**)	Clinical trial	2012-2014	40	26.4 ± 6.4 VS 27 ± 7.4	96 weeks	21 pts PEG-IFNα-2a +entecavir	19 pts PEG-IFNα-2a	–	4 (19%) in interventional group 7(37%) in control group	8 (38%) in interventional group 10(52.6%) in control group	2 (5%) patients (one in each arm)
(**Wedemeyer et al., 2019b**)	Clinical trial (HIDIT-2) (Phase 2)	2009-2011	120	38 vs 42 (20-64) vs 20-60)	120 weeks	59 pts PEG-IFN alfa-2a + tenofovir	61 pts PEG-IFN alfa-2a + placebo	–	Interventional group: 27(46%) Control group: 16(26%)	Interventional group: 18(31%) Control group: 14(23%)	22 (19%) patients (13 in internventional group, 9 in control group)
(**Anastasiou et al., 2024**)	Clinical trial (FU^$^ at 5 years of HIDIT-2) (Phase 2)	2009-2011	61	38 vs 42 (20-64) vs 20-60)	5 years	28 pts PEG-IFNα and tenofovir	33 pts PEG-IFNα + placebo	Interventional group 5 (18%) Control group 5 (15%)	–	Interventional group 21 (75%) Control group 16(48%)	
(**Etzion et al., 2023**)	Phase 2, open label, clinical trial	2016-2017	33	40 (20, 64)	72 weeks	19 pts PEG-IFN Lambda 120 mcg	14 pts PEG-IFN lambda 180 mcg	–	180 mcg group: ALT normalization 5 (36%) 120 mcg group: ALT normalization 5 (26%)	180 mcg group: BLQ^&^ (36%) 120 mcg group: BLQ^&^ 3 (16%)	13 (39%) cases of dose reduction 6 in Lambda 120 mcg and 7 in Lambda 180 mcg. 2 cases of dose interruption, one in each arm. 8 (24%) of drug discontinuation (4 in each arm)
(**Borzacov et al., 2016**)	Prospective non-randomized study	2011-2012	22	45 (18-70)	72 weeks	PEG-IFN + entecavir	–	–	Reduction of ALT (p=0.0296)	18 (81.8%) at the end of FU^£^	No reported adverse events were considered serious

^*^End of follow up; ^**^ Interferon; ^£^Peginterferon; ^$^follow up; ^&^ below limit of quantification.

Patients treated with IFN therapy typically have multiple adverse events. Commonly, the patients reported a flu-like syndrome, myalgia, fatigue, weight loss, depression and a local reaction at the site of injection (European Association for the Study of the Liver, 2017). If the reaction is defined severe (Grade III) this could lead to a drug dose reduction or discontinuation. In clinical studies (**Table 1**) the adverse drug reaction leading to discontinuation ranged from 5% to 24%.

### Real life studies on PegIFN-α-based therapy

**Table 2** shows the results of real-life studies. They were heterogeneous in type of treatment and sample size, but confirmed the data from clinical trials. In fact, in observational studies HDV RNA undetectability ranged from 7.1% to 47% (Karaca et al., 2013; Abbas et al., 2014; Keshvari et al., 2014; Bahcecioglu et al., 2015; Niro et al., 2016; Soyer et al., 2016; Wranke et al., 2017; Boglione et al., 2019) and ALT normalization at EOFU ranged from 27% to 50% (Karaca et al., 2013; Abbas et al., 2014; Keshvari et al., 2014; Bahcecioglu et al., 2015; Soyer et al., 2016). In only four studies, HBsAg loss at EOFU ranging from 0% to 22% (Karaca et al., 2013; Niro et al., 2016; Wranke et al., 2017; Boglione et al., 2019) was included. In these studies, the prevalence, type and severity of the adverse events were similar to those observed in clinical trials (**Table 2**).

**Table 2:** Observational studies investigating the impact of Peginterferon in HBV/HDV infection.

Authors Years	Type of study	Enrollment period	Sample size	Age at enrollment (mean or median and range)	Follow up	Intervention group treatment	Control group treatment	HBsAg loss at the EOFU^+^	AST/ALT Normalization at the EOFU^+^	HDV-RNA Undetectability at the EOFU^+^	Common adverse event
(**Karaca et al., 2013**)	Observational, single-center, prospective cohort study	2003-2010	32	18-65	19.5 months (± 14.4)	PEG-IFN^£^ alfa	–	0(0%)	16(50%)	15(47%)	Leukopenia, thrombocytopenia and fatigue.
(**Abbas et al., 2014**)	Observational, multicentric, prospective cohort study	2009-2011	104	30 (15-55)	72 weeks	69 pts PEG-IFN^£^-α2a 35 pts PEG-IFN^£^-α2b	–	–	ALT normalization in 38 pts (35%)	44 pts (42.3%)	Non-serious adverse events and laboratory abnormalities leading to dose modifications or discontinuation.
(**Abbas et al., 2014**)	Observational, single-center, prospective case-control study	2008-2011	20	43.7 ± 1.2	end of follow up not defined	14 pts (Group A) IFN^**^-α2a for 1 year; 6 pts (Group B) IFN^**^-α2a for 3 years.	–	–	ALT decreased at the EOT **^ç^** in responders, but increased at the end of FU**^$^**	Group A 1 (7.1%) Group B 2 (33.3%)	Anorexia, weakness, and weight loss
(**Bahcecioglu et al., 2015**)	Observational, single- center, retrospective study	2004-2012	56	47.6 ± 9.5 Vs 51.1 ± 12.4	24 weeks after EOT **^ç^**	15 pts PEG-IFN**^£^** alfa 2b	41 pts PEG-IFN**^£^** alfa-2a	–	Total patients: 19(32.2%)	Interventional group: 2(13.3%) Control group: 9(22%)	7 (12.5%) patients discontinued
(**Soyer et al., 2016**)	Observational, retrospective cohort study	2000-2011	65	36 ± 9.06 Vs 42.1 ± 11.4	Median 12 months in group 1 and 16 months in group 2	Group 1 ≤12 months of IFN therapy	Group 2 >12 months of IFN therapy (max 24 months)	–	Group 1: 4(27%) Group 2: 25(49%)	Group 1: 1 (7%) Group 2: 19(37%)	Thrombocytopenia and leukopenia 14% in group 1 (dose reduction in 1 patient), 18% in group 2 (dose reduction in 7 patients, interruption in 1 patient for 4 weeks). Flu-like symptoms 7% in group 1 and 18% in group 2. Depression 7% in group 1, 0% in group 2. Weakness 0% in group 1 and 18% in group 2. Myalgia 0% in group 1 and 4% in group 2.
(**Niro et al., 2016**)	Observational, retrospective, multicentric study		62	53 + 11	5 year after EOT^ç^	PEG-IFN**^£^** with or without NUCs^§^	–	14 (22.6%)	–	26 (41.9%)	
(**Wranke et al., 2017**)	Observational, retrospetive, single center, cohort study	1987-2013	136	37.6 (14.1- 61.3)	5,2 y (0,6-18,8 y)	52 pts IFN^**^ α (30 with NUCs^§^)	39 pts no treatment 45 pts NUCs^§^ (30 with IFN^**^ α)	8 pts in IFN^**^α 1 pt in NUCs^§^ 1 spontaneously	–	IFN^**^α vs NUCs^§^ CI: 1.3-3.0 p<0,01 IFN^**^α vs untreated CI: 1,1-2,3 p<0.03	–
(**Boglione et al., 2019**)	Observational, retrospective, single-center, cohort study	2007-2014	46	33 (27–41)	3 years	PEG-IFN**^£^** alfa 180 mcg	–	4(8.7%)		8(17.4%)	Hematological toxicity (60.9%) [including neutropenia (41.3%), anemia (375), thrombocytopenia (30.4%)], Fatigue (52.2%), Flu-like syndrome (45.6%).

^*^End of follow up; ^**^ Interferon; ^£^Peginterferon; ^$^follow up; ^ç^ End of treatment, ^§^nucleos(t)ide analogues

### PegIFN lambda-based therapy

The use of pegIFNλ is under clinical investigation for the treatment of CHD. PegIFNλ differs from PegIFN-α because it recognizes a different heterodimeric receptor complex that is largely restricted to cells of epithelial origin (liver, lung and gut) (Donnelly and Kotenko, 2010).

In a phase II Pegylated Interferon Lambda Monotherapy in Patients with Chronic Hepatitis Delta Virus Infection (LIMT-1) clinical trial (Etzion et al., 2023), 33 patients were treated with pegIFN λ (120 or 180 µg subcutaneously once weekly for 48 weeks): 7/14 (50%) of the patients treated with 180 µg had a >2 log HDV RNA decline or negative HDV RNA compared with 4/19 (21%) patients receiving 120 µg. However, only 5 of the 14 patients (36%) and three of 19 (16%) in the two groups reached the end-point (undetectable HDV RNA 24 weeks after the end of therapy).

A phase III LIMT-2 trial of pegIFNλ 180 µg for 48 weeks with 24 weeks of post-treatment follow-up is ongoing.

### Comment on PEG-IFN based therapy

According to the data shown, PegIFN therapy is unsatisfactory in terms of tolerability and virological efficacy, especially in long-term follow-up. In fact, sustained virological response was achieved in only a minority of cases, and the addition of nucleoside/nucleotide analogues has not been shown to significantly improve the outcome. However, at the moment it is the only therapy that can guarantee the loss of HBsAg, a fundamental endpoint for therapeutic success. Another point of interest is that it is not clear whether the HDV genotype can influence the outcome of therapy; in this regard, interesting are the results of a retrospective study showing that in 24 patients treated with peg-IFN, the post-treatment response was significantly better in patients infected with HDV genotype 5 (10% GT1 vs. 64% GT5, p = 0.013) (Spaan et al., 2020).

Moreover, it is important topoint out that the management of Peg-IFN-based therapy is very complex. In fact, the frequent clinical and biochemical side effects of PegIFN therapy are well known and affect different sites of the organism: dermatological manifestations, neuropsychiatric disorders, alteration of thyroid function and thrombocytopenia and a multidisciplinary approach is often necessary to manage these complications, the severity of which may be such as to lead to premature discontinuation of PegIFN treatment. Moreover, PegIFN is contraindicated not only in subjects with advanced liver disease but also in subjects with neurological, dermatological and psychiatric comorbidities as well as in the case of autoimmune diseases. In rare cases, PegIFN can be a trigger for autoimmune hepatitis.

## Bulevirtide-based therapy

Bulevirtide (BLV), previously known as myrcludex B, is a peptide entry inhibitor interfering with the interaction of HBsAg with NTCP, the recently identified receptor responsible both for HBV and HDV entry to the hepatocytes (https://www.natap.org/2023/EASL/EASL_31.htm; Wedemeyer et al., 2019a; Wedemeyer et al., 2023b; Wedemeyer et al., 2023a; Asselah et al., 2024).

The drug proved its safety and efficacy in Phase II and III trials, with the conditional approval from the European Medicine Agency in July 2020 at the dosage of 2mg/day subcutaneous injection (https://www.ema.europa.eu/en/medicines/human/EPAR/hepcludexauthorisation-details-section).

In this section, we will review all the currently available data from randomized clinical trials (RTCs) and real life studies (**Tables 3**, **4**).

**Table 3:** Results of Bulevirtide (BLV) therapy both in monotherapy and in combination therapy with PegIFNalpha derived from RTCs.

Randomized Controlled Trial name, authors, year, references	Sample size	Age, liver disease	Intervention type	Intervention time	Primary outcome: 2 log HDV RNA decline and or HDV RNA undetectable	Percentage of patients with primary outcome (and at the EOT^§^)	Percentage of subjects with the specified HDV RNA undetectable data	Combined outcome	Percentage of patients who reached combined outcome (and at EOT)	% severe adverse reactions - or discontinuations
MYR 202, (**Wedemeyer et al., 2023b**)	120	Mean 39.4 (SD 8.3) 54% compensated cirrhosis	BLV* 2mg/d+TDF**	24weeks intervention followed by 48 wks follow up	2 log decline or undetectable HDV RNA at 48 weeks follow up (EOT)	4% (54%)	EoT (end of follow up) 4% (4%)	At least 2 log HDV RNA decrease and ALT normalization at the end of follow up (at the end of treatment)	7% (21%)	0% - 0%
		Mean 40.9 (SD 9.5) 47% compensated cirrhosis	BLV 5mg/d+TDF			6% (50%)	EoT (end of follow up) 6% (3%)		3% (28%)	9% - 3%
		Mean 41.8 (SD 11.3) 53% compensated cirrhosis	BLV 10mg/d+TDF			10% (77%)	EoT (end of follow up) 3% (0%)		3% (37%)	7% -0%
		Mean 38.5 (SD 8.7) 46% compensated cirrhosis	TDF			0% (3%)	EoT (end of follow up) 0 (0%)		0% (0%)	4% - 4%
**MYR 301,** (**Wedemeyer et al., 2023a**)	150	Mean 41.8 (SD 8.4), 47% compensated cirrhosis	144 weeks BLV 2mg/d	144 weeks treatment	Combined response: virological (HDV RNA undetectable of at least 2 log decrease) and biochemical (ALT normalization) At 96 weeks follow up	At week 48: 45%	At week 48: 12%			4% - 0%
			144 weeks BLV 10mg/d	144 weeks treatment		At week 48: 48%	At week 48: 20%			8% - 0%
			Delayed 96 weeks of BLV 10mg/d	48 weeks follow up, then 96 weeks of treatment		At week 48: 2%	At week 48: 0%			6% - 0%
**MYR 203,** (**Wedemeyer et al., 2019a**)	**90**	Mean 34.1 (SD 7.0) 26.6% compensated cirrhosis	pegIFNa*** 180ug/qw^£^	48 weeks	Undetectable HDV RNA at 24 weeks off therapy follow up	0%	EoT, at week 48: 13.3%	negative HDV RNA and ALT normalization at week 72	0%	0% - 0%
		Mean 37.2 (SD 5.5) 13.2% compensated cirrhosis	2 mg BLV + pegIFNa 180ug/qw			53.3%	EoT, at week 48: 80%		46.7%	6.67% - 0%
		Mean 36.9 (SD 7.5) 26.6% compensated cirrhosis	5 mg BLV + pegIFNa 180ug/qw			26.7%	EoT, at week 48: 86.8%		13.3%	0% - 0%
		Mean 37.2 (SD 5.5) 13.2% compensated cirrhosis	BLV 2mg/d			6.7%	EoT, at week 48: 13.3%		6.7%	0% - 0%
		Mean 34.3 (SD 7.2) 13.2% compensated cirrhosis	10 mg BLV + pegIFNa 180ug/qw			6.7%	EoT, at week 48: 80%		6.7%	0% - 0%
		Mean 36.2 (SD 7.2) 13.2% compensated cirrhosis	10 mg BLV (5mg BID)			33.3%	EoT, at week 48: 46.7%		13.3%	0% - 0%
**MYR 204,** (**Asselah et al., 2024**)	**174**	Mean 41 years (SD, 8.7) 35% had compensated cirrhosis	pegIFNa qw	48 weeks treatment, 48 weeks follow up	Undetectable HDV RNA 24 weeks off therapy (Sustained Viral Response 24 at weeks); Undetectable HDV RNA At 48 weeks after the end of treatment	17%; 25%	At week 48: 21%; at week 96:	Undetectable HDV RNA and ALT normalization 24 weeks off therapy	13%, 25%	54% - 4%
			pegIFNa qw + BLV 2mg/d followed by 48wks BLV 2mg/d monotp	48 weeks IFN + BLV treatment, 48 weeks BLV, 48 weeks follow up		32%; 26%	At week 48: 40%; at week 96: 44%		28%, 22%	54%; 2% discontinuation for BLV; 6% discontinuation for PegIFN
			pegIFNa qw + BLV 10mg followed by 48wks BLV 10mg monotp	48 weeks IFN + BLV treatment, 48 weeks BLV, 48 weeks follow up		46%; 46%	At week 48: 60%; at week 96: 70%		42%, 40%	60% - 2% discontinuation for BLV; 4% discontinuation for PegIFN
			BLV 10mg/day for 96 weeks	96 weeks of treatment, 48 weeks follow up		12%; 12%	At week 48: 10%; at week 96: 12%		8%, 8%	20% - 2%

**Table 4:** Results of Bulevirtide (BLV) therapy both in monotherapy and in combination therapy with PegIFNalpha derived from real life studies.

Authors, year, (reference)	Treatment	Study design	Sample size	Treatment length	Age, Liver disease	Primary outcome	Percentage of subjects with HDV RNA undetectable data	Biochemical outcome	Combined response	Serious adverse events % - discontinuations %
de Lédinghen et al., 2021 (https://www.natap.org/2021/AASLD/AASLD_18.htm)	BLV 2mg/BLV +pegIFNa	Prospective, observational	77 monotherapy /68 +pegIFNa	12 months	41.6 med in mono group, 40.8 med in combo group. Compensated cirrhosis, adv fibrosis, F2 with ALT >2nv for 6 mnt	HDV RNA neg or >2log decrease at 12mnt: 68.3% BLV/93.9% BLV+INF	Monotherapy: 68.3%; combination: 85%	ALT neg at 12mnt 48.8% BLV/36.4% BLV+INF	39.0% BLV/30.3% BLV+INF	Grade 3-4 9%-8.8% discontinuations 2.6%-4.4%
(**Jachs et al., 2022**)	BLV 2mg/day; BLV 10mg/day. In lack of response at week 24, pegIFNa was added	Observational, retrospective	22/1	24-137 weeks	mean age: 47.9 years. 5 pts over 65 Compensated cirrhosis: 70%	HDV‐RNA decreased by >2log or undetectable in 45%, 55% 65% and 69% at 24, 36, 48 and 60 wks. 2 relapses in patients terminating treatment at >24 wks undetectable	Not specified	Normalization of ALT level: 64%, 85%, 90% and in 92% at 24, 36, 48 and 60 wks	32%, 50%, 60% 62% at 24, 36, 48 and 60 wks	1 pt needed growth factor for wbc count 4.3% - 0%
(**Jachs et al., 2022**)	BLV 2mg/day In lack of response at week 24, pegIFNa was added	Observational, prospective	7	46–141 weeks	Age range: 31 to 68 years; 1 pt over 65 Compensated cirrhosis: 57,1%	Study on suspension post undetectability; all patient undetectable: 4 relapses in BLV monotherapy, 1 patient no relapse in BLV monotherapy, 2 received pegIFN adjunction: no relapse	1 patient in BLV monotherapy; 2 patients receiving PEGIFN adjunction	Normalization of ALT level: 42.8%. 1 pt among relapses showed ALT relapse	/	28.6% - 28.6% (2 pts discontinued pegIFNa)
Fontaine et al., 2022 (https://www.natap.org/2022/EASL/EASL_44.htm)	BLV 2mg monotherapy vs BLV 2mg + pegINF	Prospective, observational	65 monotherapy/50 combination	48 weeks	43yo +/- 11/40.5yo +/- 10.9; cirrhotic 58.5%/52.%	Combined outcome (ALT negativization, HDV RNA at least 2 log decline) Monotherapy: at week 24: 17% Combination at week 24: 34%	Monotherapy: at week 24: 8% Combination at week 24: 44%	Normalization of ALT level at 24 weeks; monotherapy: 54% Combination: 35%	Monotherapy: at week 24: 17% Combination at week 24: 34%	At week 24: monotherapy: 14, combination 18 – monotherapy: 1, combination: 3.
(**Comandini et al., 2023**)	BLV 2mg	Prospective, observational	13	11 months, median	42 years [IQR 48–62], 2 pt over 65. Compensated cirrhosis	40% undetectable at 9mnt, 40% rebound at 6-9mnt	40% undetectable at 9 months	ALT reduction at 9 months 68,5%	At 6 months, 66% w/o HIV, 60% with HIV	0% - 0%
(**Comandini et al., 2023**)	BLV 2mg/day	Retrospective, observational, multicentric	114	24 weeks	Median: 47 ± 11 52% cirrhotic patient, 4.38% decompensated	HDV RNA > 2 log decrease or undetectable during follow up (38 ± 17.6 weeks): 76.3%	21.9%	ALT normalization at 12 weeks: 34.6%; at 24 weeks: 19.2%	undescribed	0% - 5.2%
(**Sandmann et al., 2023**)	BLV, unspecified dosage	Observational, retrospective	16	6 months	[Age unreported] Compensated cirrhosis: 43.8%	Decline > 2log HDV RNA: 38%	Not evaluated	Normalization of ALT level: 69%	37.5%	/
(**Degasperi et al., 2022**)	BLV 2mg/day	Observational	18	48 weeks	Median age: 48 [29-77] Compensated cirrhosis 100%	At least decline > 2log HDV RNA: 78%	23%	Normalization of ALT level: 83%	67%	0% - 0%
(**Degasperi et al., 2022**)	BLV 2mg/day	Observational	9	48 weeks	46 years [IQR 41–62] (no over 65 pt) F2 (2 pts), F3 (3 pts), F4 Child A (4pts)	HDV RNA > 2 log decrease or undetectable at 48 weeks: 85,7%	3/8 (37.5%) at 48 weeks,	ALT normalization at 48 weeks: 28,5% (all reduced, 2 started from normal values, 1 stopped)	20% (2 had already neg ALT, 1 had already undetectable RNA)	0% - 0%
(**Degasperi et al., 2022**)	BLV 2mg/day	Observational, prospective	7 (3)	24 weeks (48 weeks)	Median age: 41 ± 8.1 years Compensated cirrhosis: 57.1%	At least decline > 2log HDV RNA:71.4% (24 weeks)/66% (48 weeks), 1 virologic breakthrough	Not evaluated	Normalization of ALT level: 50% (24 weeks)/66%, 1 re-increase (48 months)	50% (24 weeks), 66% (48 weeks)	0% - 0%
(**Degasperi et al., 2022**)	BLV 2mg/day	Observational, retrospective	93	72 weeks	Median age 52 years, 100% cirrhosis, 55% varices, 22% previous ascites	at weeks 24, 48 72, HDV RNA ≥2 Log decline: 67%, 77% 75%	10%, 16%, 38% at weeks 24, 48 72, respectively	ALT normalization at weeks 24, 48 72 in 67%, 67%, 81%	at weeks 24, 48 72: 45%, 56%, 63%	0% - 0%
**Degasperi et al., 2022**)	BLV 2mg/day	Retrospective, observational, multicentric	15	23 weeks	100% cirrhosis, 66% previous ascites	decline in HDV-RNA levels by ≥2 log: 66%	Not evaluated	ALT normalization: 47%	unreported	unreported
(**Degasperi et al., 2022**)	BLV 2mg/day	Retrospective, observational, multicentric	176	Median 48, up to 96 weeks	100% cirrhosis, 46% esophageal varices, 12% previous ascites, 6% active HCC Age 50 (19–82)	at weeks 24, 48, 96, HDV RNA ≥2 Log decline: 48%, 66% and 77% undetectable in 16%, 33%, and 43%	16%, 33%, and 43% at weeks 24, 48, 96, respectively	at weeks 24, 48, 96, ALT normalization 60%, 69% and 73%	at weeks 24, 48, 96: 28%, 48% and 58%	0% - 1 pt

§ End of Treatment; *Bulevirtide; **Tenofovir Disoproxil; ***Pegylated Interferon alpha

£ once a week

### Randomized clinical trial on bulevirtide

**Table 3** summarizes the results of BLV therapy both in monotherapy and in combination therapy with PegIFN derived from RTCs.

#### RCTs evaluating BLV+PegIFN combination therapy

MYR 203 (Wedemeyer et al., 2019a) was a multicenter, randomized Phase II study in which patients received 48 weeks of either BLV or pegIFN-alpha monotherapy or combination of the two agents at different dosages for 48 weeks: pegIFNα 180 μg, 2 mg BLV+pegIFNα, 5 mg BLV+pegIFNα, 2 mg BLV, 10 mg BLV+pegIFNα and 10 mg BLV + Tenofovir Disoproxil (TDF). The primary endpoint was defined as an HDV RNA level below the limit of detection (10 IU/mL) at week 72 (24 weeks after stopping therapy) and was achieved by 0%, 53.3%, 26.7%, 6.7%, 6.7% and 33.3% of patients. Secondary endpoints were ALT normalization, combined treatment response (≥2log serum HDV RNA decline +ALT normalization), and HBsAg reduction >1log. Among the six treatment arms, ALT normalization was achieved in 10%, 53.8%, 33.3%, 23.1%, 35.7% and 35.7%; the combined response at week 72 was achieved in 0%, 46.7%, 13.3%, 6.7%, 6.7% and 13.3%; HBsAg > 1 log decline or loss was identified only in BLV+pegIFNα groups (40% for BLV 2 mg+pegIFNα, 13.3% for BLV 5 mg + pegIFNα, and 13.3% for BLV 10 mg + pegIFNα). At week 72 HBsAg loss occurred in 4 (27%) and 1 (7%) patient(s) treated with BLV2 mg or 10mg both plus pegIFNα, respectively. In MYR 204 (Asselah et al., 2024), a phase 2b randomized study, 174 patients with CHD and compensated liver disease, randomized in a 1:2:2:2 proportion were evaluated to receive pegIFNα monotherapy for 48 weeks, BLV 2mg and pegIFNα or BLV 10mg and pegIFNα for 48 weeks, both followed by 48 weeks of monotherapy with the respective BLV dose, or BLV 10mg for 96 weeks. The groups underwent a 48-week follow up after EOT. The primary endpoint was a sustained virological response at week 24 of post-therapy follow-up, defined as HDV RNA undetectabilty, which was reached by 17%, 32%, 46%, and 12% of patients, respectively. At 48 weeks after the end of treatment, HDV RNA was undetectable in 25% of the patients in the peg-IFN alfa-2a group, in 26% of those in the 2-mg BLV plus peg-IFN alfa-2a group, in 46% of those in the 10-mg BLV plus pegIFN alfa-2a group, and in 12% of those in the 10-mg BLV group. HBsAg loss was only reported in combination therapy groups, 8% of BLV 2mg + pegIFNα treated patients and 4% of BLV 10mg + pegIFNα treated patients. Finally, safety of combination therapy was comparable to pegIFNα monotherapy.

#### RCTs evaluating BLV mono-therapy

MYR 202 (Wedemeyer et al., 2023b) was a multicenter, randomized phase II study in which 120 patients with CHD, already undergoing TDF treatment for longer than 12 weeks, received either different BLV doses (2, 5 or 10mg/day), or continued to receive TDF monotherapy, for a period of 24 weeks followed by 48 weeks of follow up. The primary end-point (undetectable HDV RNA or a 2 log decline from its baseline level) at EOT was reached respectively in 54%, 50% and 77% BLV dosage groups and in 3% in the TDF monotherapy group 24 (all results in BLV groups were statistically significant compared to the TDF group). At 48 weeks of post-therapy follow up, HDV RNA rebounds were observed in all BLV groups, with the primary outcome reached in 4%, 6% and 10% of BLV treated patients and in 0% of the TDF monotherapy control group. The secondary outcome, ALT normalization, was reached at EOT by 43%, 50% 40% and 6% of patients in the respective groups, while at 48 weeks follow up by 14%, 3%, 10% and 14%; a combination of the two outcomes was obtained in 60%, 80% and 83% of patients in the BLV groups at EOT, while at the end of follow up in 7%, 3%, 3% of BLV treated patients.

MYR 301 is a phase III RCT (Wedemeyer et al., 2023a) in which 150 patients with CHD were randomized to receive either no anti HDV treatment for 48 weeks and then BLV 10mg/day for 96 weeks, or BLV for 144 weeks at 2mg/day or 10 mg/day dosage; all arms underwent 96 weeks of follow-up. Most patients in the three arms received concomitant anti HBV NUC treatment. The primary endpoint was a combined response of undetectable or at least 2log HDV RNA decrease from baseline and ALT normalization at week 48; secondary endpoints included, as a single element, virological response, biochemical response, change in liver stiffness. At week 48, BLV receiving groups showed similar combined responses (BLV 2mg, 45%; BLV 10mg, 48%), significantly greater than in the control group (delayed treatment, 2%). Virological response was reached respectively by 71% and 76% (undetectable, 12% and 20%) in the BLV2mg and BLV 10mg groups; ALT normalization was obtained in 51% and 56% of patients in the 2mg and 10mg groups, respectively, and in 12% of controls. A statistically significant improvement in liver stiffness was reached in the BLV groups. These data were confirmed at week 96: virological response was achieved in 76% in BLV 2mg arm, in 82% of BLV 10mg group, and in 90% of the delayed treatment group, with undetectability achieved in 20, 26 and 32% of patients, respectively; ALT normalization was achieved in 63, 64, and 43%, respectively. Combined response was observed in 55% of patients in BLV 2mg arm, and in 56% in BLV 10mg arm, while among patients in the delayed arm, which at week 96 had completed 48 weeks of therapy, 39% achieved a combined response (https://www.natap.org/2023/EASL/EASL_31.htm)

Another important aspect investigated in MYR 301 is the intrahepatic virological response (https://www.natap.org/2021/AASLD/AASLD_56.htm), investigated by performing liver biopsies at enrollment and 48 weeks in 27 patients in the delayed treatment group and in 21 and 31 patients in the BLV 2mg and 10mg, respectively. The specimens were tested immunohistochemistry for HDVAg and quantification of positive cells; qPCR was performed for HDV RNA, HBV DNA and RNA and host gene expression. HBV DNA and RNA levels remained low and were not affected by treatment. In BLV arms, a strong reduction in HDV RNA (-2.2 log IU/ml and -2.5 log IU/ml in the BLV 2 mg group and in the BLV 10 mg group, respectively) and in HDVAg positive cells (median -2.1 Δ log and -2.0 Δ log, in the 2 groups) was observed.

BLV has a good safety profile. An integrated safety analysis of 24-week data from MYR202, MYR203, MYR204 and MYR301 (355 patients) was evaluated and the overall incidence of participants experiencing adverse events (AE) was similar in BLV 2 mg and BLV 10 mg groups at 67.4% and 73.8%, respectively, compared to rates of 87.2% in the Peg-IFNα group and 49.4% in the control group. The rates of grade 3–4 AE and grade 3–4 laboratory abnormalities were similar in both the BLV and control groups but were higher in the Peg-IFNα group. The AE profile was similar between the BLV groups and the control group, with a few exceptions, including higher rates of headache and total bile acids increased in the BLV treatment arms. Injection site reactions occurred and were more common in the BLV 10 mg group compared to 2 mg (https://www.natap.org/2022/HDV/070122_07.htm).

BLV administration was also associated with an improvement in health-related quality-of-life. Self-reported outcomes (https://www.natap.org/2021/AASLD/AASLD_17.htm), investigated administering the Hepatitis Quality of Life Questionnaire at week 24, demonstrated an improvement in all aspects analyzed, especially for hepatitis specific limitations, hepatitis specific health in distress, and mental health, in patients treated with BLV in MYR 301 compared with the delayed treatment arm. Similarly, Buti et al. reported an exploratory analysis of EuroQol 5D visual analog scale (EQ-5D VAS) scores in patients with CHD after 48 weeks of treatment with BLV in MYR301. Patients with CHD treated with BLV 2 mg experienced improvements in QOL measured by the EQ-5D VAS at week 48. EQ-5D VAS scores significantly improved in the BLV 2 mg group compared with baseline and compared with controls at week 48, but did not improve in the BLV 10 mg group outcomes (Buti et al., 2023).

### Real life studies on Bulevirtide

In this section we will review real life studies published across Europe (Degasperi et al., 2023b; Dietz-Fricke et al., 2023a; Loglio et al., 2019; Degasperi et al., 2022; Herta et al., 2022; Jachs et al., 2022; Loglio et al., 2022; Zöllner et al., 2022; Anolli et al., 2023b; Comandini et al., 2023; Dietz-Fricke et al., 2023b; Jachs et al., 2023; Sandmann et al., 2023; https://www.natap.org/2022/EASL/EASL_44.htm; https://www.natap.org/2021/AASLD/AASLD_18.htm), in the majority of cases on BLV in mono-therapy. **Table 4** summarizes these studies.

One of the first real-life studies was done in France: thanks to the availability of BLV within an early access program in September 2019 and to a conditional marketing authorization since September 2020, 138 patients were included in the BuleDelta cohort, also known as ANRS HD EP01 (Agence Nationale de Recherches sur le Sida et les hépatites virales, for HDV code EP01). The preliminary analysis included 98 of 138 patients with available data at week 24: 54 (55%) were treated with BLV in mono-therapy, and 44 (45%) in association with Peg-IFNα; overall 74 (76%) were concomitantly treated with nucleos(t)ide analogues (NUC). The mean decrease in HDV RNA at week 24 was 1.9 ± 1.4 log IU/ml (2.6 and 1.6 log IU/ml with and without Peg-IFNα, respectively). The virologic response, i.e. a decrease in HDV RNA of at least 2 log and/or HDV RNA undetectability, at week 24 was observed in 55 (56%) patients (80% and 37%, with and without Peg-IFNα, respectively, among which undetectability of HDV RNA was reached in 44% ad 8%, respectively). The biochemical response, i.e. normalization of ALT, was observed in 36 of 98 (37%) patients at week 24 (34% and 40%, with and without Peg-IFNα, respectively) and the combined response in 25 (26%) patients at week 24 (36% and 17%, with and without Peg-IFNα, respectively) (https://www.natap.org/2022/EASL/EASL_44.htm).

Dietz-Fricke et al (Dietz-Fricke et al., 2023b). evaluated 114 patients from various German centers receiving a 2mg/day BLV therapy were followed-up for 38 ± 17.6 weeks: 59 patients had cirrhosis, of which 4 were Child-Pugh B and 1 was Child-Pugh C; 55 patients had previously received Peg-IFNα therapy. A virologic response defined as an HDV RNA decline of at least 2 log or undetectable HDV RNA was observed in 87/114 (76%) cases over the mean observation time of 38 ± 17.6 weeks, 21.9% reached undetectability of HDV RNA; however, in 11 cases a virologic breakthrough (>1 log-increase in HDV RNA after virologic response) was observed. Cross sectionally, at 24 weeks of treatment, 19/33 patients (58%) had a virologic response, while three patients (9%) did not achieve a 1 log HDV RNA decline. No patient lost hepatitis B surface antigen. Alanine aminotransferase levels improved even in patients not achieving a virologic response, including five patients who had decompensated cirrhosis at the start of treatment. In 6 patients of the 114 analyzed, BLV was discontinued, in one of these because of a lack of response; no serious adverse events were described.

In the Sandmann study (Sandmann et al., 2023), a German study conducted in a Hannover center, 16 patients undergoing 6 months of BLV therapy were included, 7 with compensated cirrhosis, all receiving concomitant NUC therapy. HDV RNA declined in all patients during treatment; at 6 months, 38% (6/16) showed ≥ 2 log HDV RNA decline from baseline levels and 11 patients (69%) normalized ALT levels, while 4 patients (25%) achieved both outcomes.

The data from Austrian patients are described in two studies by Jachs et al, in 2022 and 2023 (Jachs et al., 2022; Jachs et al., 2023). In the first study (Jachs et al., 2022), 23 patients were treated for various lengths of time, with the peculiarity of adjunction of Peg-IFNα treatment in patients fit for treatment and who did not reach virological outcome within 24 weeks. Interesting are the data on the efficacy and tolerability of BLV in cirrhotic patients. According to literature data, BLV monotherapy seems to be safe and useful in patients with severe CHD, as in patients with cirrhosis and significant portal hypertension for which treatment with Peg-IFNα is contraindicated and BLV, therefore, represents the only therapeutic option. Certainly, a good safety and tolerability profile is even more important if antiviral therapy is to be administered long-term in patients with advanced liver disease.

Loglio et al (Loglio et al., 2019; Loglio et al., 2022). described the first 3 European patients with HDV-related compensated cirrhosis who were treated with BLV 10 mg/day for 48 weeks as a compassionate therapy. Liver function tests, bile acids, and virological markers were monitored every 4 weeks. During treatment, HDV RNA levels progressively declined from 4.4 and 5.6 logs IU/ml to undetectability in 2 cases, and from 6.8 log copies/ml to 500 copies/ml for the other patient. Alanine aminotransferases normalized after 20, 12 and 28 weeks, respectively, and there was a significant improvement in features of portal hypertension, in liver function tests and in alpha-fetoprotein levels with a good safety profile.

Anolli et al (Anolli et al., 2023b). evaluated in a retrospective multicenter Italian real-life study the safety and efficacy of BLV 2 mg monotherapy up to 72 weeks in HDV patients with compensated cirrhosis; 93 patients were included: median age was 52 years, 52% males, median liver stiffness measurement (LSM) was 17.4 kPa, 55% had varices, 22% had previous ascites, 53% were IFN-experienced, 97% under NUC treatment. Median ALT levels were 79 U/L, albumin 3.9 g/dL, platelets 70 x 103/mm3, HDV RNA 5.2 log IU/mL, Child Turcotte Pugh (CTP) score A in all patients. A virological response (undetectable HDV RNA or ≥2 log decline *vs.* baseline) was achieved by 67%, 77% and 75% of patients at weeks 24, 48 and 72, respectively, HDV RNA becoming undetectable in 10%, 16% and 38%. At the same timepoints, ALT normalization was observed in 67%, 67% and 81% of the patients while a combined response (undetectable HDV RNA or ≥2 Log decline *vs.* baseline ALT normalization) was achieved by 45%, 56% and 63% of patients. Besides ALT, significant on-treatment declines were also observed for AST, GGT, IgG (p<0.001 versus. baseline), while albumin values increased (p=0.02). BLV was well tolerated and no patient discontinued treatment for adverse events, an asymptomatic increase in bile acids occurred in all patients. During BLV treatment, liver decompensation occurred in one patient, *de-novo* HCC in two, three underwent liver transplantation and one died because of BLV-unrelated causes.

Interesting also are the results of Save-D study (Degasperi et al., 2023a; https://www.who.int/news-room/fact-sheets/detail/hepatitis-d), a retrospective multicenter European study, showing the virological and clinical outcomes of patients with HDV-related compensated cirrhosis treated with BLV monotherapy. In this study, 176 patients receiving BLV monotherapy up to 96 weeks (median follow-up: 48 (8–96) weeks) were included. At enrollment the median age was 50 (range 19-82) years, 59% were men, median ALT 77 (23–1, 074) U/L, liver stiffness measurement (LSM) 18.3 (6.4-75.0) kPa (30% with LSM >25kPa), platelets 89 (17-330) G/L (80% with PLT<150 G/L), 100% CTP score A, 9% HIV-positive, 46% with esophageal varices, 12% with a history of previous ascites, 6% with active HCC, 91% on NUC. Rates of virological responses i.e. HDV RNA undetectable or ≥2-log decline versus baseline) at week 24, 48 and 96 were 48%, 66% and 77%, respectively; HDV RNA was undetectable in 16%, 33%, and 43% patients, respectively. Biochemical response, i.e. ALT <40 U/L, was achieved by 60%, 69% and 73%, respectively, while rates of combined response were 28%, 48% and 58%, respectively. The cumulative risk of *de-novo* HCC at week 96 and decompensation was 5.9% (95% CI 2-12%) and 2.4% (95% CI 1-5%) (n=2 ascites, n=1 variceal bleeding), respectively. Six (3%) patients underwent liver transplantation (n=4 for HCC, n=2 for end-stage liver disease) and 2 patients died of BLV-unrelated causes (pneumonia and intestinal infarction). The overall cumulative survival rate was 93% (95% CI 88-98%) at week 96. In this study, according to the safety profile, the authors reported that bile acids significantly increased, 10% patients reported mild and transient pruritus. Injection site reactions occurred in 3% of cases; 1 patient discontinued BLV due to a grade 3 maculopapular rash with mild eosinophilia.

Degasperi et al. (Degasperi et al., 2022) evaluated 18 patients treated for 48 weeks with BLV 2mg/d, all undergoing nucleos(t)ide treatment, for HDV-related severe cirrhosis; in fact all BLV patients had significant portal hypertension. During 48 weeks of BLV monotherapy, HDV RNA declined by 3.1 (0.2-4.3) log IU/ml (p <0.001 vs. baseline), becoming undetectable in 5 patients (23%). Overall, a virological response, defined as undetectable HDV RNA or a ≥2 log decline versus baseline, was observed in 14 (78%) patients and a virological non response, defined as <1 log decline of viremia at week 24, was observed in 2 (11%) patients. ALT decreased to 35 (15–86) U/L (p <0.001 versus baseline), normalizing in 83% of patients. At week 48, 12 patients (67%) achieved a combined virological and biochemical response. Regarding markers of liver function, albumin and cholinesterase levels significantly improved, so 4 out of 5 Child-Pugh A6 patients at baseline improved to Child-Pugh A5 at week 48. Regarding the 14 patients achieving a virological response, the liver stiffness measurement decline was significant (16.4 kPa at baseline vs. 14.1 kPa at week 48, p = 0.03). None of the patients developed decompensating events (including ascites, encephalopathy, bleeding) or *de novo* or recurrent HCC during the study period. None of the patients discontinued BLV and no symptomatic adverse effects were reported, and bile acid increase was not symptomatic. The results of this study showed a good safety profile of BLV in patients with severe cirrhosis.

Dietz-Friecke et al (Dietz-Fricke et al., 2023a). showed data on off-label BLV monotherapy in 15 Austrian, Italian and German patients with decompensated liver disease. CTP stage B and C or with clinical signs of decompensated cirrhosis were included. Virological response (decline in HDV-RNA levels by ≥2 log) was observed in 66% of patients, in 47% of patients ALT normalization was observed, in 4 patients improvement of liver function was observed from CTP B to C, in 4 patients an improvement in ascites was observed. According to the safety profile the results showed that in one patient a worsening in liver function to CTP C was observed, in one patient there was further decompensation, in 3 subjects BLV was terminated at liver transplantation.

All these data, although on a small number of patients, support the antiviral efficacy of BLV in decompensated cirrhosis and encourage to investigate in clinical trials the long term efficacy and safety profile of BLV in this highly vulnerable population.

### Comment on Bulevirtide based therapy

The innovative therapy with BLV, both in monotherapy and combination therapy, shows interesting results, although it is necessary to wait for a longer follow-up.

As regards the safety profile, the data from both clinical and real life trials are encouraging: BLV treatment was well tolerated without any serious drug-related adverse events or treatment discontinuations (https://www.natap.org/2023/EASL/EASL_31.htm; https://www.natap.org/2021/AASLD/AASLD_56.htm; https://www.natap.org/2022/HDV/070122_07.htm; https://www.natap.org/2021/AASLD/AASLD_17.htm; Wedemeyer et al., 2019a; Buti et al., 2023; Wedemeyer et al., 2023b; Wedemeyer et al., 2023a; Asselah et al., 2024). A minority of patients complained of mild symptoms like fatigue, nausea, headache, dizziness or showed a reduction in platelets or white blood cells; adverse reactions at the injection site were mild, transient and only occasionally required specific treatment (Behrendt et al., 2022; Schwarz et al., 2022). Since BLV inhibits the bile acid transporter function of NTCP as expected, a transient increase in total bile acids was reported in all studies without clinical significance (Hagenbuch and Meier, 1994; Stieger, 2011). Also encouraging were the data from an Italian study (Degasperi et al., 2022) on patients with HDV-related cirrhosis and significant portal hypertension showing that 48 weeks of BLV 2 mg/day monotherapy was safe in patients with compensated cirrhosis and clinically significant portal hypertension. Moreover, in a clinical case described by Anolli et al (Anolli et al., 2023a), a patient with compensated cirrhosis and esophageal varices treated for 76 weeks with 10mg/d, and with 5mg/d for the following 68 weeks, a combined response was observed, platelet count and liver stiffness improved (liver stiffness also improved at the end of follow up even when compared with end of therapy evaluation, in which ALT was already negative) and small esophageal varices without red color sign, at the start, were not detected at the end. In conclusion, BLV is generally well-tolerated with fewer side effects compared to Peg-IFN, since it has been shown to be effective in long-term use, even in patients with advanced compensated cirrhosis.

As regards the virological and clinical efficacy, data from clinical trials and real word studies demonstrated significant positive effects of BLV-based therapy, although the heterogenicity of the treatment, virological endpoints and the length of therapy, make comparison of results difficult. In this innovative therapeutic approach, it is necessary to clearly identify the outcomes of virological response. It is well known that the efficacy of an antiviral is assessed by the undetectability of viral genome. Although the mechanism of action of BLV is peculiar in that it acts by saturating NTCP, an entry receptor of HDV, therefore without a role on HDV replication, the virological efficacy should be assessed by the undetectability of the viral genome. However, many studies, both RTCs and real-life studies, considered a virological response that also includes a two-log reduction in HDV viremia and it is not always possible to extrapolate the pure data of viral suppression. Among RTCs undetectability is part of the primary outcome in MYR 204 and MYR 203 with better results when BLV is associated with pegIFNα compared to PEG IFN monotherapy and BLV monotherapy, while in in MYR 202 and MYR 301 the data is extractable from secondary outcomes (**Table 3**). Most of the real-life studies (**Table 4**) include BLV monotherapy and data on viral suppression are present in Fontaine et al., 2022 (https://www.natap.org/2022/EASL/EASL_44.htm), in Visco Comandini et al., 2023 (Comandini et al., 2023), in de Lédinghen et al., 2021 (https://www.natap.org/2021/AASLD/AASLD_18.htm), Dietz-Fricke et al., 2023 (Dietz-Fricke et al., 2023b), Degasperi et al., 2022 (Degasperi et al., 2022), Zollner et al., 2022 (Zöllner et al., 2022), Dietz-Fricke et al., 2023 (Dietz-Fricke et al., 2023a) in Degasperi et al., 2023 (Degasperi et al., 2023b).

However, several questions are again open: for example the role of Peg-IFN, the possible identification of stopping rules during treatment, the duration of BLV monotherapy and the causes of non-response to BLV therapy.

The role of the addition of Peg-IFNα to BLV remains unclear since the data are few and especially from RTCs with short follow-up after stopping treatment; thus, Peg-IFNα therapy adjunction, given the high burden of its side effects but its potential benefit, was one of the most difficult clinical decisions. MYR 204, recently published in the New England Journal of Medicine (Asselah et al., 2024), the most ambitious RCT in terms of primary endpoint, demonstrated how a sustained viral response could more easily be achieved when Peg-IFNα therapy was added to BLV alone. The few real world studies seem to be coherent with RCTs: in de Lédinghen et al (https://www.natap.org/2021/AASLD/AASLD_18.htm). a virological response after 12 months BLV therapy seems to be more likely when therapy with Peg-IFNα is added. In Jachs, et al, 2022 (Jachs et al., 2022), Peg-IFNα therapy was added when no significant benefit was observed after the first 24 weeks of therapy, with good results in terms of virological outcome.

However, real-world studies, such as those of de Lédinghen and Jachs, while providing useful insights into real-world clinical settings, cannot influence guidelines. These data should therefore be interpreted as complementary, while the main decisions remain guided by the results of the RCTs.

It is not easy to compare the different strategies used in RTCs and Real life studies, as they are heterogeneous in terms of therapeutic protocol and virological outcomes. The data show that the association of BLV and peg-IFN improves the virological outcome, but the optimal length of these treatments has not yet been established furthermore and unfortunately, not all patients tolerate peg-IFN. Therefore, it will be necessary to test head to head, or combine together, in specific studies, the drugs currently available for HDV, peg-IFN and BLV, with experimental drugs characterized by other mechanisms of action, hoping to soon have a poly-pharmacological treatment available that is able to suppress HDV replication.

Another open question is to identify in the first months of treatment the patients that will not respond. However, today we have no stopping rules. To this regard Degasperi et al. offer interesting attempts to identify baseline and on-therapy predictors of response evaluating 49 HDV patients with compensated cirrhosis treated with BLV monotherapy 2 mg/day up to 96 weeks, enrolled in a single-center study (Degasperi et al., 2023b). The results of this study showed that different baseline and on-treatment HDV RNA cut-offs may predict virological, biochemical and combined response rates. In particular, for these patients clinical and virological variables were assessed at baseline and every 8 weeks. Virological response rates (undetectable or HDV RNA ≥2 log decline versus baseline) showed 71% at week 24, 84% at week 48, 75% at week 72 and 80% at week 96, respectively; biochemical response rates were 57%, 68%, 81% and 67%, while combined response was 46%, 63%, 63% and 66%, respectively. The Authors showed that baseline HDV RNA <1000 IU/ml was associated also with virological response at week 48 (p = 0.007), 72 (p = 0.04) and 96 (p = 0.04), while it did not predict combined response at any timepoint. HDV RNA <1000 IU/ml at week 24 predicted virological response (p = 0.03), combined response (p =0.02) and ALT<1.5 ULN (p = 0.02) at week 96, but not biochemical response.

Also very interesting are the results of a study that evaluated whether continued therapy could give benefits to patients without virological response after 24 weeks of treatment with BLV. In particular Lampertico et al (https://www.natap.org/2023/AASLD/AASLD_59.htm). evaluated this considering 114 CHD patients who completed BLV monotherapy for 96 weeks in the MYR301 and MYR204: after 24 weeks of therapy, 34/141 (24%) had a partial response (PR), defined as HDV RNA decline ≥1 but <2 log10 IU/mL, and 15/141 (11%) had a non-response (NR), defined as HDV RNA declines of <1 log10. Of the 34 PR patients at week 24, 25 (74%) had a virological response (HDV RNA negativity decline of ≥2log) by week 96. Of the 15 NR patients at week 24, 7 (47%) had a virological response and 3 (20%) had PR by week 96. Moreover, a higher proportion of NR at week 24 achieved a virological response at week 96 among those receiving BLV 10mg (4/5, 80%) versus BLV 2mg (3/10, 30%) and among week 24 NR or PR, the mean baseline HDV RNA did not predict viral response at week 96. These results provide evidence for continuing BLV therapy despite early (24week) suboptimal virologic responses.

Another open question remains the duration of treatment. On this point the paper by Jachs, Panzer et al. evaluating patients who discontinued BLV treatment is interesting (Jachs et al., 2023). The seven patients enrolled (age, 31-68 years, four with cirrhosis) discontinued BLV treatment after 46-141 weeks of treatment after long-term HDV suppression (time of HDV-RNA negativity: 12-69 weeks) and were followed-up for 14 to 112 weeks. One patient was lost to follow-up before the 24th week. HDV-RNA became detectable again in three patients within 24 weeks, while another patient subsequently showed a recurrence of HDV-RNA after almost 1 year; HDV-RNA remained undetectable in two patients treated with BLV + peg-IFN- α2a. BLV was reintroduced in three patients after 13-62 BLV-free weeks and was well tolerated and all patients achieved a virological response again (Jachs et al., 2023)

Finally, it would be important to identify the causes of non-response to BLV. On this point the paper by Hollnberger et al. is interesting (Hollnberger et al., 2023). The authors, in patients who were non-responders (20 patients) or who experienced virologic breakthrough (1 patient) to BLV in MYR202 and MYR301 study, performed deep-sequencing of the BLV-corresponding region in HBV PreS1 and of the HDV HDAg gene, and *in vitro* phenotypic testing at baseline and week 24. No amino acid exchanges associated with reduced susceptibility to BLV within the BLV-corresponding region or within HDAg were identified at baseline or at week 24. These results suggest that BLV has a high barrier to resistance, and this would make it a suitable drug for long-term treatment, but long-term surveillance for resistance needs to be performed with other studies to confirm these results.

## Treatments in development

Deeper knowledge of the HDV life cycle offers prospects for new therapies able to act with different mechanisms than BLV or PEG-IFN.

According to current knowledge, other inhibitors of the NTCP receptor, required for viral entry, inhibitors of the farnesyl transferase enzyme, which mediates prenylation of the large delta antigen protein that is essential for HDV virion morphogenesis, and new drugs that interfere with HBsAg production might be additional therapeutic tools against HDV and could be key points for future therapies.

A way to inhibit NCTP is represented by the anti-pre-S1 domain monoclonal antibody (HH-003). This antibody targets the preS1 domain of HBV and HDV preventing the binding with NTCP, blocking the infection and re-infection of hepatocytes. In a phase 2 trial including 9 participants with HBV/HDV co-infection the HH-003, administered once every two weeks for 24 weeks, showed the ability to reduce serum HDV RNA levels below the limit of detection or a ≥2 log10 IU/ml decline from baseline in 77.8% of participants at 24 weeks, and in 66.7% after 24 weeks of follow up. Alanine-aminotransferase normalization was detected in 60% of patients at 24 weeks, and in 40% after 24 weeks of follow up. Combined response was highlighted in 60% at 24 weeks and in 40% after 24 weeks of follow up (Wang et al., 2023).

Small interfering ribonucleic acid (siRNA) are other molecules that offer prospects for new therapies, interfering with HBV or HDV genome. The SOLSTICE trial is a phase 2 trial investigating the efficacy and safety of VIR-2218, an siRNA targeting HBx region of HBV genome, and VIR-3434, an Fc-engineered human monoclonal antibody targeting the conserved antigenic loop of HBsAg in patients with HDV infection on treatment with nucleoside analogues (tenofovir or entecavir) with undetectable HBV DNA. Three groups were defined: Cohort 1a (5 patients) including VIR-2218; Cohort 1b (6 patients) including VIR-3434; Cohort 2c (6 patients, 2 from cohort 1a, 4 from cohort 1b) including VIR-2218 plus VIR-3434 combination therapy. Virological response was defined as lower limit of detection (<LLOD) when HDV RNA < 63 IU/mL and limit of detection (<LOD) when HDV RNA was < 14 IU/mL at 12 weeks. The best virological responses were found in patients in Cohort 2c (combination therapy) with 100% of patients with <LLOD and 80% of patients with <LOD. Few treatment-related adverse events were observed and were all grade 1 or 2 (https://www.natap.org/2023/AASLD/AASLD_44.htm).

In a phase II, non-randomized study (Bazinet et al., 2017), 12 patients with CHD were treated with nucleic acid polymers (NAPs), that interact with HBsAg leading to degradation of intracellular HBsAg. The patients initially received NAP REP 2139 (500 mg intravenously/once weekly) monotherapy for 15 weeks, followed by a lower dose (250 mg) in combination with Peg-IFNα for an additional 15 weeks, followed by Peg-IFNα monotherapy for 33 weeks. At end of therapy, 9/12 (75%) treated patients had undetectable HDV RNA, which was maintained in 7/11 (64%) at 1 year of follow-up. All 12 patients experienced at least one adverse event during treatment: two (17%) patients experienced anemia, eight (67%) neutropenia, and ten (83%) thrombocytopenia. Five (42%) patients had raised alanine aminotransferase levels, four (33%) had raised aspartate aminotransferase levels, and two (17%) had increased bilirubin concentrations. Four (33%) patients had a serious adverse event, and 12 (100%) patients had treatment-emergent laboratory abnormalities.

Another target of treatment may be the inhibition of prenylation. Lonarfanib (LNF) is a farnesylation inhibitor, acting on the large delta antigen, able to avoid the wrapping of HDV nucleoprotein with HBsAg. After a proof-of-concept study, in which the drug was administered for 4 weeks with a reduction in serum HDV RNA, Lonafarnib dosage was tested in two Phase II trials. **Table 5** summarizes the results on LNF studies.

**Table 5:** Randomized- controlled trials on Lonafarnib-based therapy.

RCT, Phase	Authors, year, references	Sample size	Age, cirrhosis	Intervention type	Treatment time	Follow up time	Primary outcome	Number (%) of patients who reached primary outcome	Secondary outcome	Number (%) of patients who reached secondary outcome	% severe adverse reactions - or discontinuations
**LOWR 1, II** (**Degasperi et al., 2023b**)	(Yurdaydin et al., 2018)	3	59 - mean	LNF* 200 mg BID**	12 weeks	/	Decrease in viral load				0% - 0%
		3	40 - mean	LNF 300 mg BID	12 weeks	/					0% - 0%
		3	46 - mean	LNF 100 mg thrice‐daily	5 weeks	/					0% - 33.3%
		3	41 - mean	LNF 100 mg BID + pegIFNα*** 180 μg weekly	8 weeks	/					0% - 0%
		3	46 - mean	LNF 100 mg BID + RTV^£^ 100 mg day	8 weeks	/					0% - 0%
**LOWR 2, II** (https://www.natap.org/2023/AASLD/AASLD_59.htm)	(Yurdaydin et al., 2022)	55	23 to 66	At least 75 mg LNF BID + RTV	12 weeks	/	Virologic: HDV RNA ≥2 log decline or undetectability	6 (46%) – in all oral regimens			18.7% -
			27 to 70	25 or 50 mg LNF BID + RTV	24 weeks	/					10.5% -
			29 to 59	25 or 50 mg LNF BID + RTV + pegINFa weekly	24 weeks	/		8 (89%) – with IFN addition			15.7% -
**D-LIVR, III** (**Hollnberger et al., 2023**)	Etzion et al., 2023 (https://www.natap.org/2023/AASLD/AASLD_59.htm)	405	42.9 (10.8) – 26%	LNF 50mg BID + RTV 100mg BID	48 weeks	24 weeks	Combined: HDV RNA ≥2 log decline and ALT normalization	18/178 (10.1%)	HDV RNA ≥2 log decline and ALT normalization taken singularly	26/178 (14.6%) – 44/178 (24.7%)	8% - 19%
			41.4 (11.5) – 26%	LNF 50mg BID + RTV 100mg BID + PegIFNa				24/125 (19.2%)		40/125 (32%) – 43/125 (34.4%)	14% - 18%
			42.3 (11.0) – 27%	PegIFNa				5/52 (9.6%)		19/52 (36.5%) – 6/52 (11.5%)	10% - 21%
			45.7 (10.9) – 29%	Placebo				1/52 (1.9%)		2/52 (3.8%) – 4/52 (7.7%)	4% - 19%

*Lonafarnib; **Bis in day (twice daily); ***Pegylated Interferon alpha; £ Ritonavir

In the LOWR-1 (LOnafarnib With and without Ritonavir in HDV– 1) trial (Yurdaydin et al., 2018a) 15 patients underwent one of the following regimens: LNF 200 mg twice per day (*Bis in Day*, BID) for 12 weeks, LNF 300 mg BID for 12 weeks, LNF 100 mg thrice‐daily for 5 weeks, LNF 100 mg BID in combination with Peg-IFNα 180 μg once weekly for 8 weeks and LNF 100 mg BID with ritonavir (RTV) coadministration 100 mg once‐daily for 8 weeks. In this study, larger doses of LNF in monotherapy obtained better responses but with a higher burden of side effects; this latter defect was prevented when ritonavir was added, improving the pharmacokinetic properties of the drug.

Based on these results, the LOWR 2 trial (Yurdaydin et al., 2022) enrolled 19 patients treated with a 12-week regimen of high‐dose LNF (at least 75 mg BID with Ritonavir 100 mg/day), 24 patients with 24 weeks low‐dose LNF (25 or 50 mg bid + RTV), and 12 patients with combination low‐dose LNF (25 or 50mg BID+ RTV) and Peg-IFNα weekly injection. The primary endpoint (at least 2 log HDV RNA reduction or undetectability at the end of treatment) was reached in 46% of low and 89% of high dose regimens, with better results described when Peg-IFNα was added. Gastrointestinal tract-related adverse events were described in 49% and 22% of high and low dose patients; these results seemed to encourage further experimentation in IFN based combination therapies.

In the currently ongoing D-LIVR (Delta Liver Improvement and Virologic Response) phase III trial, of which the preliminary 48-week results are now available (https://www.natap.org/2023/EASL/EASL_78.htm): 405 patients were randomized to receive 48 weeks of treatment with either RTV boosted LNF (50mg+100mg BID), or RTV boosted LNF (50mg+100mg BID) with Peg-IFNα, or Peg-IFNα monotherapy, or placebo. Median age of patients enrolled was 42.7 years; all patients enrolled had a low HBV DNA load (< 20IU/mL), ALT levels between 1.3 and 10 times normal values, HDV RNA of at least 500 IU/mL, HDV genotype 1 in 96% and cirrhosis in the 27% of cases. The primary endpoint, defined as a combined normal ALT serum values and at least 2 log HDV RNA reduction from baseline, was reached in 10.1% of the oral therapy group and in 19.2% of the oral + IFN group, both with a statistically significant advantage on placebo. In the Peg-IFNα monotherapy group, the primary endpoint was achieved in 9.6% of patients. Factors associated with not-reaching the primary endpoints were an HBsAg level of at least 1000 at baseline and male gender, while age below or equal to 45 years was associated with reaching the primary endpoint. At paired liver biopsies, a histologic endpoint (at least 2-point improvements in Histology Activity Index with no worsening in Ishak fibrosis score) was also evaluated: a statistically significant advantage was reached by the combination therapy group. Under the safety point of view, discontinuations were similar in all groups, while 14% of patients receiving combination therapy had at least one serious adverse event, against 8% of patients receiving oral therapy, 10% of those receiving IFN alone, and 4% placebo.

To conclude, LNF, mainly in combination with Peg-IFNα, appears to be a candidate for finite therapy of patients with CHD, but data on long-term follow-up are needed.

## Conclusion

HDV-related chronic hepatitis seems to have become a potentially curable disease, a statement that was unthinkable a few years ago. We have old and new weapons at our disposal. **Table 6** shows our interpretation of the therapeutic regimens that can be used in patients with HDV-related chronic hepatitis or HDV-related cirrhosis.

**Table 6:** Personal interpretation of therapeutic options according to liver disease.

Liver disease	Contraindication to Peg-IFN	Standard therapy
**Chronic hepatitis or compensated cirrhosis without esophageal varices**	no	Peg-IFN+Bulevirtide 2mg for 48 weeks
	yes	Bulevirtide 2mg
**Compensated cirrhosis with esophageal varices**	Yes	Bulevirtide 2mg
**Decompensated cirrhosis**	yes	Unknown Bulevirtide 2 mg is an option

The old weapons are PegIFN alpha and now lambda. PegIFN alpha, for which there are more data, appears to be an excellent combination therapy in patients for whom it is not contraindicated, both for BLV, data supported by important clinical trials and real-world studies, and probably also for Lonarfanib, although in the latter case the results are not yet definitive as the studies are fewer. However, data on long-term follow-up are needed.

For subjects for whom PegIFN is contraindicated and who have advanced liver diseases, monotherapy with BLV can be used and it cannot be excluded that in future it may be associated with antiviral drugs with complementary mechanisms of action. In this setting other studies are needed to establish the stopping rules during treatment, the duration of BLV therapy and for whom finite treatment may be possible.
